# Selections and Abstracts

**Published:** 1903-08

**Authors:** 


### SELECTIONS AND ABSTRACTS.

### MEDICINE AND SURGERY IN THE PHILIPPINES.*

I have been requested to talk to-night about the diseases of the Philippine Islands and of some of the gunshot wounds which came under my observation during my two years and a half tour of duty in the Archipelago.

My personal experiences would be of little interest to any one but myself, so I will try to confine my remarks to subjects of some general professional interest, and in order not to take up too much time I will only speak of some of the more interesting phases of the most conspicuous diseases met with.

I arrived in the Philippines in the latter part of the year 1899, and the regiment I was with was sent almost immediately to the island of Samar, as part of General Kobbe's expedition, which was the first expedition of Americans to Samar and Leyte. We landed at Catbalogan, the capital of Samar, and the headquarters of the insurgent government. Here we met with considerable resistance, and in the affair which took place we had several killed and a number wounded. In addition to our own injured we had a lot of native injured prisoners to be cared for, and this occurred throughout my experience, the prisoner patients greatly outnumbering the soldier patients. From this time on I had plenty of interesting surgical work. One of the best houses left in the town was assigned as the hospital, and I fitted it up with the excellent equipment we had been provided with for that purpose. Here I received all the wounded which were able to be transported, from all over the island.

The hospital was a nine-room frame house with corrugated iron roof and hardwood floors. Two large rooms were used as wards, the two containing twenty-five beds. The others were used as dispensary, operating-room, dressing-room, kitchen, dining-room,

*Read by Walter D. Webb, M.D., Assistant Surgeon U. S. A., before the New York Post-Graduate Clinical Society.

etc. I had one hospital steward and seven hospital corps men to act as nurses, orderlies, litter-bearers, etc. At first I was the only surgeon present, but later, as the work increased, I was sent an assistant, Contract Surgeon Frederick D. Branch, of Albany, N. Y., who proved himself a very valuable man.

We were well equipped with all the instruments required for general surgical work, dressings in great abundance, a large Arnold sterilizer, suture materials sterilized and contained in hermetically sealed tubes and also in bulk, antiseptics and all the drugs used in ordinary medical and surgical practice in liberal amounts. We even had a microscope with the accessories necessary to examine fresh and stained specimens. The Government was very liberal in providing us with everything needed. All that was required was a little foresight to get a proper requisition in and receive your goods before the want of them became urgent. Many volunteer surgeons lacked this foresight and later blamed the army for what was really their own fault.

The larger number of gunshot wounds which came under my observation and care occurred in natives and were made by the small bore, high velocity bullets, from our own army rifle, the Krag-Jorgensen, which has a caliber of 30mm. diameter.

The many wounds which I had to treat among our own men were made with a great variety of weapons, bullets of nearly all calibers, bolos and spears.

The Mauser and the Remington were the guns that the greatest number of insurgents who had guns at all were armed with, but among their ranks could be found a great variety of small arms, shotguns and rifles.

The Mauser is approximately the same caliber as our rifle, and like the Krag, the bullet is encased in a nickel-steel jacket. The Remington carries a large lead bullet encased in a brass jacket, which usually goes to pieces on contact even with soft tissue. Before going to the Philippines I read all the theories I could find, accounting for the character of wounds made by the small bore, high velocity bullets at a given range. I soon found I could not tell the range by the character of the wound nor foretell the character of the wound which would occur at a given range. There are so many influencing factors involved, size of projectile, velocity, tissue involved, angle at which it impinges and probably many others, that with our present knowledge, I do not believe we can accurately predict that at a given range we will have an explosive effect with the production of a definite kind of wound and at another given range a penetrative effect with its definite wound.

However, as a rule, at all ranges, wounds involving the cranium or compact cancellous tissues were accompanied by extensive comminution, and wounds involving the soft tissues were, almost always, perforating in character, with very small wounds of entrance and exit.

This was the rule, at all ranges, but exceptions for which I could not account were not uncommon. I saw a number of accidental wounds of the hands and feet where the muzzle of the gun was either in contact or within a few inches of the injured member, and in these cases a small perforating wound was the usual result unless the metacarpal or metatarsal bones were involved, and then there was usually extensive comminution with forcing of the fragments outward and making a large wound of exit. Also a number of wounds made at ten to twenty yards, in native prisoners, shot while attempting to escape, failed to show the explosive effect described for this range, unless the cranium was involved. Again, I saw a number of wounds made at one thousand yards or more which also failed to show the characteristic wounds described for that range. Three natives who were shot at a distance of fourteen hundred yards sustained perforating wounds with small wounds of entrance and exit, two through the chest and the third through the thigh.

Wounds of the head, as I have said, were accompanied by extensive comminution, especially at the wound of exit, and the mortality was very high as compared with the wounds made by the old bullets. Most that I saw resulted in immediate death, and the explosive effect was well demonstrated at all ranges. A Chinaman shot at fifteen yards with a Mauser recovered from a wound of the cranium with the loss of a considerable portion of the right side of the vertical portion of the frontal bone.

Wounds of the thorax were in practically all cases perforating, with small wounds of entrance and exit. An uneventful recovery, with few symptoms other than the expectoration of a small amount of blood, was the rule, unless a large vessel was involved, in which case death occurred from hemorrhage, usually on the field.

When the wound of exit was large, which sometimes occurred from the bullet striking a rib, there was a collapse of the lung and the formation of a hemopneumothorax.

Several wounds of the chest were of special interest illustrating the difference between the modern small caliber jacketed bullet and the old large, soft lead bullet.

Private C. of the Forty-third Volunteer Infantry was shot at about seventy-five yards with a Mauser. The wound of entrance was very small, about the size of the diameter of the projectile, which is twenty-nine mm. It was situated in the eighth interspace in midaxillary line of left side.

The wound of exit was about three times as large as that of entrance, caused by the bullet striking the seventh rib, one inch post to post axillary line, on the right side. The right lung was collapsed and the pleural cavity contained a large amount of blood. It was a marvel to me how the bullet could pass through both lungs from side to side along the tract followed in this case without hitting some large vessel or vital structure. In addition to the gunshot wound of the chest this soldier sustained fourteen bolo wounds, and yet he made a recovery.

Wounds of the abdomen were highly fatal, and I believe in operating at once when there are perforating wounds of one of the hollow viscera, even with the most meager outfit. I do not believe we are justified in allowing the case to go without an attempt to close the wounds in the stomach or intestines.

I had a large number of compound comminuted gunshot fractures of the long bones to treat. If the blood supply of the extremity distal to the wound was good I tried to save the arm or leg, even though the comminution was extensive. I removed the detached bone and left those large fragments which had periosteal attachments above and below; these I tried to mould into postion and then applied some form of immobilizing dressing.

The first case I treated in this way was an extensively comminuted fracture of the upper third of the thigh made by an old-fashioned, brass-jacketed Remington ball, fired at long range. It occurred in an adjoining town and I was sent for to amputate the extremity, but on finding that the blood supply was unimpaired by comminution, I decided with many misgivings to try to save the limb. I was influenced in coming to this decision by the fact that the fracture was so high that a disarticulation through the hip-joint would have been necessary. As I had never done this operation at that time, besides having very poor facilities and meager assistance at the small town, I was not at all anxious to attempt it. I enlarged the wound of entrance, removed the lodged ball and loose fragments, many of which were very large, moulded the fragments with periosteal attachments into place, made a posterior drainage and immobilized the extremity. The result was a good, useful limb, but with considerable shortening.

After this first case I saved a number of arms and legs where extensive comminution with loss of bone had occurred, which I believe I would have amputated had I not seen this result.

I only amputated three lower extremities for gunshot wounds. In two the circulation was so destroyed that gangrene of the foot followed, and in the third the shattering of the lower extremity of the femur was so extensive that an amputation was indicated beyond a question.

Had I to go over the experience again I think I would amputate a little more frequently, as I have thought since that some of the extremities saved were not of enough value to warrant the long tedious treatment. In gunshot wounds of the knee-joint the lives of two soldiers could have been saved by early amputation. Three men were brought on the same day to the hospital with gunshot wounds involving the knee-joint. One, Private F., of the 43d Volunteer Infantry, was shot with a Mauser, and the other two, Private C. of the 23d Infantry and Private M., 43d Infantry, with large soft bullets. Private F.'s wound was perforating, with small wounds of entrance and exit. The bullet passed through the head of the tibia, then through the joint and made its exit just internal to inner margin of patella.

With an aseptic dressing and immobilizing with plaster bandage the patient made an uneventful recovery and returned to duty with no impairment of the functions of the joint. The other two had large wounds of entrance and exit and were infected on coming into my hands. One of the bones entering into the formation of the joint was in each case comminuted. I opened the joints thoroughly, removed bone fragments, established drainage with a number of rubber tubes and irrigated the joints twice daily with normal salt solution. Private M. did well shortly after cleaning the joint out and in a short time I had him up in a wheel-chair with a normal temperature. Private C. ran an irregular temperature for several weeks and several pockets had to be opened in the thigh, but finally his temperature became nearly normal and he improved greatly in general condition. At this stage I was relieved by a volunteer surgeon and I turned the two cases over to him. Later I heard he had amputated in both cases. Private M. died on the table and Private C. died shortly after the operation. Although I feel confident that the lives of these two men could have been saved by proper attention to the dressings and without amputation, I decided that unless I could see the case through to the finish or knew who I was turning the case over to, I would amputate early in infected wounds of the knee-joints.

I cannot speak in too high terms of the value of the first-aid package. Many instances came under my notice in the Philippines where the application, by a soldier, of the dressing, was the means of saving the wound from infection and in this way undoubtedly saving life. All armies to-day provide every officer and enlisted man with a first-aid package. Ours consists of a piece of gauze, a triangular bandage and a large safety-pin, all sterilized and wrapped first in oiled paper then in rubber cloth and sealed at the ends. All soldiers are instructed in the use of the dressing and instructions are printed on the outside. Recruits will throw away their packages or use them for sore feet or to clean their guns, but not so the man who has had some active campaigning; he has learned the value of the package and will wear it strapped to his cartridge belt until everything else is gone. We found a limited number of first-aid packages, made in Germany, in use among the insurgents in Samar.

After fourteen months in the Southern Islands I was ordered to Manila and assigned to one of the General Hospitals. There were four hospitals in the city at this time, the First, Second and Third Reserve Hospitals and Santa Mesa, but shortly after my arrival the Second Reserve was discontinued and Santa Mesa only took venereal and tubercular cases.

These hospitals were very well equipped, the Government gave us every thing asked for in the way of surgical and medical equipment. The operating-room was excellent at the First Reserve and would compare favorably with those in the large modern hospitals in the cities of the United States. Nurses were in abundance, but all the Manila hospitals were under-equipped with medical officers.

For one year I did the operating at the Third Reserve Hospital, and then at my own request was put in charge of the Army Pathological Laboratory where I remained until my tour expired. The surgical work in Manila was of quite a different character to that in the provinces. It consisted largely of the remote effect of gunshot wounds, besides the general surgery similar to that seen in any hospital, hernia and appendicitis, both of which are so common among young men, appendicitis especially so in the tropics, where there is so much intestinal disease.

The aseptic results in the Manila hospitals were very good. I had less than one per cent. of infections in wounds intentionally made. I saw personally to the sterilization of all dressings, gowns, etc., and prepared my suture material, with the exception of the use of some of the hermetically sealed tubes prepared by St. John Levens.

We wore rubber gloves, and gauze caps to prevent the falling of hair and dandruff, besides, in very hot weather, a gauze sling under the chin to prevent the falling of sweat on the field of operation. The same germs which cause wound infections in the States occur in the Philippine Islands, under the same conditions and with about the same degree of frequency. Tetanus is quite common among the natives, who neglect small wounds of the hands and feet. And tetanus neonatorum is a common cause of mortality among the native infants.

Of all the diseases which attack our army in the Philippines the intestinal are by far the most serious. Of these dysentery stands first, both in point of causing mortality among and in invaliding the soldiers. About 15 per cent. of all admissions to the Manila hospitals was for dysentery.

The varieties seen are acute and chronic amebic dysentery, acute and chronic specific dysentery and acute and chronic colitis from other causes.

The amebic dysentery is more frequently slow in its onset and runs a chronic course. This, however, is not the invariable rule by any means, as we see many cases with a sudden onset terminating fatally or in recovery in a short time or going on to a chronic course. The differential diagnoses between the two varieties of dysentery were made by the aid of the microscope, the amebic being very easily demonstrated by examining a smear from the fresh stool. The specific dysentery gave the serum reaction with the Shiga bacillus, usually after the fifth or sixth day. The technique was similar to that used in the Widal reaction and the dilution used most frequently was one to forty. There are undoubtedly a number of varieties of amebæ in the Philippines, not all of which are pathogenic. They can be found in the stools of soldiers in perfect health. There is a great deal of work yet to be done with the amebæ. Those seen engulfing red blood-cells were generally considered to be pathogenic.

The pathology of the two varieties of dysenteries differs markedly. In the amebic cases there are situated in the colon, with maximum intensity either in the sigmoid or cecum, deep ulcers with undermined, overhanging edges. The mucosa and submucosa are greatly thickened, and two of the ulcers will frequently communicate by tortuous tracks under the mucosa. In the autopsies I did, and saw, the maximum intensity was oftenest in the cecum, and in a number of cases the ulcers extended some distance up the ileum. The picture in specific dysentery was quite different. The colon is intensely congested, injected and ecchymotic and covered with superficial erosions; the mucosa is not markedly thickened.

The treatment of dysentery was very unsatisfactory. That which I found most successful was absolute rest in bed during the acute stages, withdrawal of all food for the first twelve to twenty-four hours, and a free evacuation of the bowels with magnesium sulphate, frequently repeated. If begun early this cut many of the cases short and brought about a prompt recovery. The use of opium, bismuth and other drugs which prevent free evacuation of the bowels was avoided both in the acute and chronic stages, as I believe that free drainage from the ulcerating surfaces is of the greatest importance. All drugs by the mouth were unsatisfactory with the exception of magnesium sulphate.

The ipecac treatment was not what we had hoped for it, yet I saw some real benefit from its use in some cases, when vigorously carried out.

Local treatment by high irrigations with quinin 1-5000 and stronger. Tannin .5 per cent. solutions, nitrate of silver in weak solutions and so on through the great list which has been recommended was carried out very extensively and conscientiously. Especially the irrigation with solutions of quinin. I finally gave them all up for the normal salt solution, as I came to believe that the very real benefit derived from irrigation of the large bowel was due more to the removal of the discharges and ridding the bowel of the foul fermenting toxin-laden contents than to any specific action or power to destroy the organisms by the solutions used.

When preparations of opium were used it was usually to control pain or aid in retaining large doses of ipecac; in both cases I used morphine hypodermatically. The serum treatment of specific dysentery was not tried.

The complication of amebic dysentery most frequently seen was abscess of the liver. In about 20 per cent. of all cases dying with dysentery liver abscesses existed. In twenty-three autopsies which I did in cases dying with dysentery there were eleven liver abscesses. Of these seven were multiple and four single. The diagnosis in the early stages is very difficult, as they do not usually present the picture described in the text-books until they have existed for some time and are very extensive or are associated with one of the pyogenic organisms. I believe that they are single in the early stages and later become multiple, but unfortunately when we see them they are more frequently multiple. I had two cases at the same time which illustrate very well the very different clinical picture which cases of liver abscess, both of which contained the ameba dysenteriæ, can present. Private T. of the Signal Corps was brought in on a stretcher extremely emaciated, with an irregular septic temperature accompanied by profuse sweating. Leucocytes 38,000 per cu. mm.

Two large abscesses were opened and the patient lived two weeks. On autopsy multiple abscesses were found throughout both lobes. The second case, which was admitted at the same time, Corporal H. G., Company M, of the 23d Infantry, walked into the hospital weighing 156 pounds, which was within a few pounds of his normal weight. His cheeks were florid and he looked almost in perfect health. His temperature was only slightly above normal. Leucocytes were normal. He had a history of having had dysentery one year previous but had none on admission. On examination he was found to have a large swelling in the epigastrium, slightly to the right of the median line, which, according to his statement, had been present for several months. This turned out to be a large liver abscess from which I evacuated 1600 cc. of characteristic pus, containing many active amebæ. The patient made a rapid recovery and returned to duty. Had the swelling not been present I do not think any one would have suspected his having a liver abscess.

Leucocytosis in the cases of amebic liver abscess which I saw was not a very valuable symptom, and I doubt if it is present without a mixed infection.

The amebæ were sometimes difficult to find on first opening the abscess, but later, when the walls began to slough, they appeared in the discharge. In two cases which I saw the abscess had ruptured into the bronchi and the amebæ appeared in the sputum.

Next to dysentery the intestinal parasites are the most serious of the diseases with which we have to deal. They do not directly cause many deaths, but as they so frequently complicate the more fatal diseases they are a potent contributing cause of mortality. Aside from this they are probably one of the most frequent causes of invaliding the soldiers to the United States and resulting in a great loss to the Government.

The following result of stool examination made during one month at the Army Pathological Laboratory will show the frequency of the various parasites, and also the great value of the microscope in tropical medicine.

| | |
|---|---|
| Stools examined | 268 |
| Ameba dysenteriæ | 47 |
| Ova ankylostoma duodenale | 28 |
| Embryo ankylostoma duodenale | 28 |
| Embryo strongyloides intestinale | 2 |
| Ova ascaris lumbricoides | 19 |
| Ova tricocephalus dispar | 25 |
| Balantidium coli | 1 |
| Tricomonas intestinale | 36 |
| Circomonas intestinale | 6 |

The ankylostoma duodenale, it will be seen, is one of the most frequent and is also the most serious of the parasites to the American. These cases were admitted to the hospital with the greatest variety of diagnoses, the most frequent among which were anemia, nephritis and endocarditis. Most of the cases of so-called tropical anemia are cases of ankylostoma. The symptoms of an advanced case are marked anemia. Red blood-cells often reduced to 100,000 or less per cu. mm. Hemoglobin often to 20 per cent., an anemic cardiac murmur, edema of the lower extremities and a muddy yellow complexion. Constipation is more frequent than diarrhea in uncomplicated cases. The ova can be readily found in the stool. The treatment was, unlike that of dysentery, very satisfactory. Large doses of thymol effect a speedy cure, although it may have to be repeated a number of times to rid the patient finally of the parasite.

A number of different intestinal parasites are often associated in the same case. Private C. R. was admitted to the hospital with acute dysentery and with a history of long-continued diarrhea. His stools on examination in the laboratory were found to contain ameba dysenteriæ, ova of ankylostoma duod. and ova tricocephalus dispar, and in addition to this he vomited two adult worms, ascaris lumbricoides, while under treatment. He entered hospital September 3, and was returned to duty cured September 18. Except for his prompt recovery this is not a very unusual case.

Many cases were treated in the hospital with the diagnosis of sprue. This is a condition following dysentery, chronic diarrhea or infection with one or more of the intestinal parasites, which results in a general atrophic condition of the mucous membrane and glandular structures of the entire alimentary canal from the mouth to the anus. The mouth is sore, the mucous membrane glazed, and often covered with small ulcers. The stools are large, frothy, fermenting masses. The advanced cases are extremely emaciated, they run a subnormal temperature and present very much the same picture as does a marasmic child which is dying from starvation from lack of assimilation. The only treatment which seems to be of any great benefit is a transfer to a colder climate.

Malaria is very prevalent in the Philippines. The benign tertian and the estivo-autumnal parasites are both common; the quartian parasite is rare. The disease responds very readily to quinin and differs in no way from that seen in the southern parts of the United States. I usually employed quinin in solution with aromatic sulphuric acid and gave it in moderate doses, from 5 to 10 grains three times a day, except in very urgent cases and when it could not be retained by the stomach. In these cases I gave the hydrochlorate hypodermatically in doses of from 2 to 3 grains.

Recurrences were very frequent unless the treatment was kept up for a month or two after disappearance of all symptoms.

The two cases of hemoglobinuric or black water fever I saw were very different from the symptomatic hematuria or hemoglobinuria which I saw so frequently in cases of malaria. They both terminated fatally within forty-eight hours, one with convulsions and complete suppression of urine and the other with almost complete suppression. The urine passed was black and on examination this was found to be due to free hemoglobin. Both patients became deeply jaundiced shortly before death. The malarial parasite could not be demonstrated in either the peripheral or splenic blood, in the marrow of the long bones or in sections from the spleen. The spleen was very slightly if at all enlarged. The kidneys were intensely congested with hemorrhagic spots under the capsule and scattered through the cortex. The case with complete suppression was given large doses of quinin hypodermically, while the other case received no quinin at all. Only the first case gave a malarial history.

Typhoid fever is not at all common, and when I left the islands it seemed to be growing less so than during the early days of occupation. Not a single case occurred among the troops I served with in Samar and Leyte during the fourteen months I was with them, and this seemed remarkable to me after our experience with typhoid in 1898 in the South and in the West Indies. There are very few flies in the Philippines as compared with the United States and the West Indies, and I have thought this the possible explanation of the fact that typhoid does not spread in the Philippines, where all other conditions are apparently so favorable.

I expected to encounter a number of tropical fevers of undetermined type, and undoubtedly such fevers do exist, but it was very rare to meet with a case which could not be diagnosticated, as one of our described diseases, by clinical, laboratory or post-mortem methods.

Malta fever, although not one of the prominent diseases of the Philippines, is by no means rare. I saw several cases, in all of which the diagnoses were confirmed by the serum reaction.

Dengue is one of the commonest diseases in the islands. Few Americans who spend any length of time there escape it. It is not at all dangerous, but, as I know from personal experience, it is not at all pleasant. Its principal points of interest to me were its obscure etiology and the great variety of eruptions seen in different cases. Macular, papular, sometimes vesicular and even pustular eruptions were seen. Beriberi is very common among the natives, but very rarely attacks the American, although it may occur in the adjoining house. Puerperal women seem to be very susceptible to the disease and the mortality is very high among them.

Smallpox is very prevalent. In the provinces mild cases are often seen in the village streets. When it occurs among our soldiers it seems to be of a specially virulent type and the mortality has been very high. Of great interest is the excellent work which has been done since the American occupation. Vaccine farms were started at once and compulsory vaccination enforced, with the result of greatly diminishing the disease, especially in the city of Manila, where the percentage of cases has fallen from a very high figure to a low one. Bubonic plague seldom attacks the American in the Philippine Islands, and in those few cases which have occurred the victims were of a class which are under no restraint and who had taken up their abode in the shacks among a low class of natives. The attendants at the plague hospital and those who have handled the dead have not contracted the disease. All this does not prove that plague would not be a very serious disease if it once got into the crowded tenement districts of the cities of the United States, especially San Francisco, where there are so many Orientals. The Manila Board of Health has been very energetic in disinfecting and burning infected districts and in destroying rats, but as yet they have been unable to stamp the disease out entirely.

I left the islands before the cholera epidemic had gained any marked headway and saw only a few cases.

Tuberculosis is among the first rank of the diseases of the Philippines. It is a scourge among the natives, and naturally many Americans, whose general condition is apt to be lowered by the heat, by intestinal diseases, malarial and other troubles, fall victims to tuberculosis in one of its many forms. Its course in the American is extremely rapid, far more so than at home. No one can realize how rapidly pulmonary or joint tuberculosis can prove fatal who has not seen it in the tropics. After seeing a number of cases die while under conservative treatment. I amputated a number of times in cases of joint tuberculosis, as the only means of saving life.

Elephantiasis is either absent or very uncommon. During my tour of duty I did not see a single case, and I have not been able to find any one who did see cases in the Philippines. This seems queer, as the filaria nocturna is quite common. I found it in the blood of a number of Chinamen and natives whom I examined. It is contrary to the authorities on tropical medicine, who say that when filaria nocturna is present elephantiasis always exists. Mycetoma or fungus-foot of India is not very uncommon and on casual view may be mistaken for elephantiasis.

Yaws, which is known as Boté among the Visayians, is very common in Samar and Leyte, where most of the natives are attacked with it at some time during their lives. It is a repulsive sight to see the natives often walking about the streets covered with the granulomata which are present in this disease, discharging a thin yellowish pus and bleeding on the slightest contact. They all seem to recover and do not take the disease very seriously.

Venereal diseases are not more common than under similar conditions in the United States and they follow about the same course. When a soldier who is already debilitated or is suffering from intestinal or some other disease contracts syphilis, it is only natural that it should run a more virulent type.

Away from the large cities and seaports venereal disease was not very prevalent among the natives.

Leprosy is quite common, and every town throughout the southern islands has its quota of victims in various stages of the disease. In some places they are segregated, while in others they live at their homes among their families. Manila has a large leper hospital, San Lazero, where all known cases are sent. A leper colony is at present being established.

In all hot climates skin diseases are rife and the Philippines are no exception. Dhobee itch is a term used loosely by the laity and by a great many of the profession to include a variety of skin diseases. The term originated in India where the word dhobee means washerman. It is a form of tinea infection which was supposed to be conveyed by the washermen.

The commonest skin disease seen in the Philippines is an infection with one of the ringworms, of which there is a great variety.

Pemphigus contagiosa and impetigo contagiosa are very common. Ulcers and boils are seen in great numbers and in all classes, but are especially common among the bare-footed community.

DISCUSSION.

*Major William H. Arthur, Surgeon, U. S. A.:* My own service in the Philippines led me into a different line of duties and experiences from those so interestingly outlined in Dr. Webb's paper.

I was twice on duty in the Archipelago. First in November, 1899, when I arrived there in command of a hospital ship to take home some of the débris of General Otis's army. The second time when, after a few months' service in China with the Relief Expedition, I reported for shore duty, and was assigned to the charge of the base hospital at Dagupan, Province of Pangasinan in northern Luzon.

Though in this hospital (and subsequently in the principal military hospital in Manila, of which I took charge in December, 1901) we saw a few recent gunshot wounds, as the active campaign was practically over in Luzon we encountered more of the remote effects of small arm projectiles on the body. These presented no points of particular interest, being treated exactly as they would be at home. Our surgery consisted principally of hernias, appendicitis cases and liver abscesses, of which a large number following amebic dysentery were seen and operated upon with a result of about 40 per cent. mortality. All, or nearly all, the fatal cases were those in which the dysentery persisted, or in which there were multiple abscesses. Our general results in surgical cases were very good, and I can say this without undue egotism, as the majority of the operations were performed by my subordinates, under my direction, and not by myself, my administrative duties keeping me many hours at my desk, when I should much have preferred to be in the operating-room.

Under, in some cases, what the civil surgeon would consider very unfavorable conditions, we secured good aseptic results by simple cleanliness. At the Dagupan hospital (which was a fine old Spanish school building), the operating-room consisted of the end of a ward partitioned off with bamboo poles over which sterilized cotton sheets were stretched. These were changed daily. Two rooms of this kind were provided, one for septic and one for clean cases. The water supply at Dagupan consisted of a shallow well. Water was drawn from this, promptly boiled and filtered, and carried to the operating-room in large tin vessels supplied by the quartermaster's department.

At the Manila hospital the operating-rooms were much better, but the results were as good (I mean as far as securing asepsis was concerned) in the Dagupan establishment. Our greatest enemy was dysentery. This I say advisedly, notwithstanding the fact that we had Asiatic cholera and plague to deal with. As Dr. Webb says, we were disappointed in the ipecac treatment of dysentery, though the East Indian surgeons I met in China were enthusiastic about it.

The question of amebæ is still in doubt. That is, we do not know yet how to tell the pathogenic ameba from the perfectly harmless kind. Dr. Musgrave describes fourteen varieties found in stools. Those which are found to contain red blood-corpuscles are supposed to be the most suspicious, but the question needs furthed investigation. Musgrave gave doses of salts to perfectly healthy soldiers, who had been in the islands a year or more and found amebæ in stools of 68 per cent. None of these men had ever had at that time any symptoms of dysentery.

The few cases of dysentery caused by the Shiga bacillus infection (acute "specific" dysentery, it was called) were uniformly and rapidly fatal. I saw only seven cases in my own practice. Out of twenty-nine at the First Rescue Hospital in Manila, twenty-three were fatal.

The operation recently advocated and performed here in New York, with promising results (I mean utilizing the vermiform appendix for irrigating the large intestine), was not tried while I was in the Philippines. The thorough irrigation of the lower bowel by high injections with the patient's elbows on the floor, his thighs and legs being supported on a low table built for the purpose, seemed to insure very fair irrigation of the descending and transverse colon.

Typhoid fever did not become very prevalent in the Philippines. I saw only twelve cases in the two years I spent in Philippine military hospitals. This failure to spread I attribute to the fact that there are, for some reason, few flies in the islands.

The cases that did occur followed the usual course of the disease, unmodified by the climate.

Plague occurred in very few cases among Americans, and was uniformly fatal. The Chinese and Filipinos probably were more exposed from the fact that they habitually go barefooted. The only two cases that came to my hospital in Manila (only officers, soldiers and civilian employees of the army were treated in this hospital) occurred in teamsters who had been living in native huts.

Asiatic cholera appeared in the spring of 1902, and many cases occurred in Manila and the provinces, especially among the natives. The Chinese escaped almost entirely. This was explained by the greater cleanliness of the Chinese and the fact that they drink very little, except water in the form of tea, under ordinary circumstances. Many cases were brought to the First Reserve Hospital but were promptly transferred to a special cholera hospital as soon as their nature was determined. Most of the cases I saw were of the "cholera sicca" type, the intestinal discharge not being sufficiently copious to account for the rapidly supervening shriveling of tissues, coldness of extremities and fatal termination. The cholera infection is the most virulent and the toxemia the most intense I have ever seen in any disease. Fortunately, its spread among intelligent people can be easily prevented. The health department found great difficulty in controlling cholera among the ignorant natives, and the priests did much to render its efforts nugatory, by telling the people that they were being poisoned by the Americans. Smallpox, which had been a scourge, was controlled by vaccination, and I saw only a few cases in the Dagupan and Manila hospitals.

What Dr. Webb says of tuberculosis corresponds exactly with my experience. The disease runs a very rapid and destructive course in the Philippines. Indeed, this is true of all pyogenic processes. Wounds may be made to heal as promptly as at home by great care in securing asepsis, but once they become infected the destruction of tissue is very rapid and destructive, more so than I have ever seen it in similar cases at home.

*Prof. Samuel Lloyd:* I do not know why I should be called upon to talk about medicine and surgery in the Philippines, for I have never been there.

There are a few things that Dr. Webb suggested that have appealed strongly to me. In the first place, having heard Dr. Webb before, and, in fact, being responsible for his appearance before you to-night, I have had the opportunity of talking over with him some of these subjects. There is one thing in particular that we are in accord with, and that is in regard to the medical superstitions that have been handed down from time immemorial, yet when we come to the actual clinical experiences we find that they do not exist. Still they are referred to in the text-books and are occasionally met with in private practice. I am glad that, in the Philippines, they find the same condition of things. Text-books show that, in their treatment of certain subjects, the authors copy blindly what has been said in older books, without proving the truth of the statement.

With regard to liver abscesses, while we see but very few, yet I have had four in my experience. All four were instances of single abscesses, and all but one recovered. The question brought out by the doctor was very interesting in that it showed the amebic conditions corresponded exactly with the conditions produced by other infections. Absence of infection from débris is what we expect. The progress of the disease is in the walls or at the periphery. No germ will live on its own débris, but it crawls out where it can get something to feed on. This applies to the ameba as well as other pathogenic germs. That is why in attempts to destroy germs in such instances little result will follow unless we dig away the lining membrane and get into the tissues beyond the point of degeneration.

Another point that has interested me very much was the handling of gunshot fractures, the comminuted fractures, because it bears out thoroughly my idea of treating compound fractures. I believe that we should carefully render the parts aseptic, replace the pieces of bone that have periosteum attached to them, remove the fragments that have been denuded of periosteum, and then place the limb in the best possible position for recovery. Such a course of procedure will be productive of good results. Both Dr. Webb and Dr. Arthur referred to cases that later gave bad results; I think that those cases had the injuries near joints and in the cancellous structure, and may have been subjected to those influences which lead to osteomyelitis. I have now at St. Francis Hospital a gunshot wound of the knee-joint which has entirely healed. The X-ray shows nothing to account for the conditions present, and yet this patient has a useless and painful knee. The question arises, "What is it due to?" The probabilities are that we shall have to amputate it. It seems to me that the attempt at saving the limb in order that we might possibly get a good limb was well worth while. A good many such limbs have been saved only to be amputated later, but a great many more have proven useful and satisfactory. It is time enough to amputate when it is demonstrated that it is useless.

The absence of infection in so many cases in the Philippines to me is very wonderful, especially as it occurs in a country where they neglect the general rules of sanitary living. One would expect septic diseases to spread very rapidly there, and, as a consequence, operative work would be followed more frequently by infection. It is remarkable that they obtain such results. It may be that the absence of flies has something to do with it, in that they cannot carry the streptococcic infection from one patient to another.

*Prof. Ogilvy:* I should like to ask a question with regard to bullet wounds of the abdomen. Dr. Webb referred to a case in which the patient died from peritonitis and I would like to ask him if most cases were operated upon. I should also like to know the percentages of recovery with and without operation.

*Dr. Walter D. Webb:* All cases that I saw of gunshot wounds of the abdomen with perforation of the intestines or stomach, if not operated upon, died either immediately or soon after their infliction from peritonitis. Only two cases recovered, and in both there were perforations of the intestines or stomach. One of these cases I operated upon, stitching two holes in the colon. The other case was operated upon by another.

*Dr. Peterson:* I think the doctor should congratulate himself upon having had his experience in the Post-Graduate Hospital with its great facilities in teaching medicine, surgery and pathology, and I am sure he appreciates the training he has obtained here. We, as alumni of the hospital congratulate ourselves upon sending out such a man to do such work in the Philippines.

*Dr. Webb:* I strive to be a general practitioner and do all sorts of work, and I certainly appreciate the training I have obtained here, which has helped me to do something in various lines of work.

*Dr. Hexamer:* It has been stated that typhoid fever was not very prevalent in the Philippines and I am at a loss to know why. It struck me that, especially in the Department of No. Luzon, every post-surgeon had a sterilizer and every post-surgeon labored to see that the sterilizer was working. If not working, the chief surgeon of that department was to be informed of that fact by wire. I think this is the reason why there was so little typhoid fever. I landed the same time Dr. Webb did, only he went south and I went north; my experience has been the same as his.

With regard to Dhobee itch, it covers a multitude of sins, like charity; but most of these cases of itch were simply and solely the results of contamination of sudamina by the finger-nails. Besides that most cases of dhobee itch were cases of impetigo contagiosa, and a great many cases occurred among the natives. These cases were found in the section of country I was in.

There was another thing about the gunshot wounds in the Philippines which interested me very much. Give a Filipino a rifle with a range of 1000 yards, he was sure that everything on that rifle had to be used, and, consequently, he used it. If he should have used a rifle with a range across a room, the Filipino would use it at a range of one mile. I know of an instance of L Co., 12th Infantry, lying in the open, in a rice paddie, within twenty-three yards of the insurgent's trench; they remained there fifty-five minutes and not a man was hit. I saw one cartridge, the use of which was not permitted by the rules of modern warfare. I saw the Filipino coming in conflict with the ladrone. He had a brass jacket on the cartridge. Where the bullet went I do not know, but I do know that I dug the jacket out of the man's shoulder.

Dr. Webb entirely left out syphilis and gonorrhea in his remarks. I know it has been stated by members of faculties of schools, and prominent ones too, that syphilis among the Filipinos was a curable disease, *i. e.*, would disappear entirely. My experience with syphilis and venereal diseases generally was obtained in this school, whose survivors are Bangs, Otis, and many others of equal reputation. I have seen syphilitic cases among the natives with complete perforation of the hard palate just as clean and round and of same nature as seen in children three or four years old, and I have seen them die, and I do not think it is time to make the claim that syphilis is different there from what it is here. I could not see any difference. I remember a patient, a clergyman in charge of a church, who had secondary syphilis as bad as ever I laid my eyes on. In the town I refer to there were probably twenty syphilitic subjects. The Filipinos get married when they are only ten or twelve years old. As soon as they are big enough to toddle they get married. Syphilis in the Philippines is not an uncommon disease. I hope that Major Webb and Dr. Arthur will add something on this subject. Gonorrhea is the same there as it is on Twentieth street. Among the Filipinos there seems to be a lack of the power of repair. A man may run a bamboo thorn in his hand and suppuration will follow which will have absolutely no relation to the severity of injury received at all. I once saw a Filipino who had been to a chicken-fight such as takes place every Sunday there. It seems that the chicken had struck him with his spur just above the knee-joint. The injury was poulticed when I first saw him with what looked like spinach. This I removed and washed the part and sent him to the hospital. Within twenty-four hours that man had an infection of a very septic nature, with the inguinal glands involved; within another twenty-four hours he was dead. I wanted to open the part up and wash it out but the family objected to it. As Dr. Webb has already stated, army surgeons, above all other men, must be men of expediency and must be able to do something without anything. At Manila everything possible was to be had for the asking, from quinin pills to a binocular microscope. But when back in the country sixteen or eighteen miles from any railway you are upon your own resources. Medical men in the United States Army have proven that they are equal to any emergency. As General Sherman once said, "War is hell," but I know that for the medical men in the Philippine Islands it is two hells.—*The Post-Graduate.*

---

**This Journal and Atlanta Semi-weekly Journal one year for $1.00 cash in advance. No checks. Strictly to new subscribers.**

### THE TREATMENT OF PUERPERAL ECLAMPSIA WITH A REPORT OF CASES.*

In obstetrics, perhaps more than in any other department of medicine or surgery, we are frequently confronted with the sudden onset of complications which seriously endanger the life of both mother and child. No one of these complications is more serious or more to be dreaded than eclampsia. Fortunately it is not a frequent complication, occurring only once in 250 to 400 cases, according to different observers, but the mortality is high, ranging from 20 to 30 per cent. for the mothers and 50 per cent. for the infants. Nothing but our best efforts will be sufficient to overcome such a mortality.

If we would treat a disease satisfactorily we must know its cause. While this is not definitely known in the case of eclampsia, the many theories advanced in the past as to its cause have gradually been giving way to the belief that the convulsions are due to the presence of some toxin in the blood. The failure of the kidneys to perform their functions suggested the possible etiologic relationship of urea, a factor which is no longer considered as the sole cause. The failure of the kidneys, however, does aid in the accumulation in the blood of the causal toxin, whatever it may be. These are questions that future investigations must settle. To-night I wish to limit myself to a discussion of the treatment of eclampsia.

Eclampsia may occur during pregnancy, labor or the puerperal state and its treatment must be modified accordingly, but, without waiting for the onset of convulsions, there should be vigorous prophylactic treatment as soon as any symptoms occur to warn us of danger. These symptoms are rapid pulse with high tension, headache, gastric disturbances, difficulty in seeing, swelling of the feet, hands and face, decrease in the amount of urine and the presence of albumin. All of these are not present in each case, and it is well to remember that because the albumin is absent we are not necessarily free from danger. The mere examination for albumin is not enough. We must watch for the other symptoms. An

*Read by F. S. Clark, A.M., M.D., Cleveland, before the Academy of Medicine of Cleveland, January 16, 1903.

estimation of the solids excreted during twenty-four hours is especially valuable, though it is difficult to make with accuracy. If these are found to be decreasing, appropriate measures should be taken to restore the normal conditions. Sometimes many of the above symptoms may be present without convulsions occurring, or, on the other hand, most of them may be absent and convulsions occur suddenly. As in all diseases, there are cases which are so rapidly fatal that nothing can be accomplished. There are also cases that are so severe that most thoroughly prophylactic measures will fail to stop the occurrence of convulsions, but it will probably postpone them to a time when the danger will be much less, for the mortality is lower during and after labor than before.

Accurate information regarding the origin of the toxin would be valuable in directing the prophylactic treatment. Experience shows that beneficial results follow the restriction of food just as in uremia, so that an exclusive milk diet is best. In the milder cases, if it is possible to tell which are to be such, slight additions could be made of some of the least harmful foods. Winckel says that light meats could be given, but this hardly seems wise.

Our efforts, as far as possible, must be directed toward freeing the system of those toxins which are already formed. The failure of the kidneys indicates our best guide to the line of treatment that should be adopted to accomplish this result. For diuresis, mild alkalin waters are the best and should be taken freely. The potassium salts are apt to be irritating, and it is better not to use them. In addition to this, stimulation of the functions of the intestines and skin is of course invaluable. The bowels should be moved freely every day by the use of some laxative if needed. Phosphate of soda in small doses is effectual and especially good because of its mild action on the liver. Occasionally it may be wise to take a cathartic dose either of this or of one of the other salts. High injections of salines aid by flushing out the bowels. A warm bath should be given each day to keep the skin in good condition. When these means fail and the symptoms grow worse the production of labor is indicated, and it is not wise to delay unnecessarily.

As has been said, prophylactic treatment sometimes fails, while in other cases no precautions have been taken and we are suddenly confronted with convulsions. Now, more than ever, most vigorous treatment is necessary to save our patients. First and most important of all we must eliminate the toxins from the system, and second, we must control the convulsions. If the convulsions occur before or during labor the uterus should be emptied at once. Occasionally there are cases in which the convulsions can be controlled and the cause removed without terminating labor, but in attempting this we are running great risks, for if we find our efforts to remove the cause and so to control the convulsions fail, we have lost valuable time and greatly diminished the chances of our patient's recovery. It would seem far better to risk losing a premature child with correspondingly brighter prospects of saving the mother than to increase the risk of losing the mother with very little decrease of infant mortality. The uterus should be emptied by manual dilatation of the os or incisions of the cervix, if need be, the child being delivered with forceps. The growing tendency on the part of the surgeon to recommend Cesarean section instead of the more reasonable obstetric operations cannot be recommended. The results following this operation for eclampsia are anything but flattering, and the skillful obstetrician can obtain far better results by the old methods.

After the uterus is emptied the treatment is the same as when the convulsions do not occur till labor is normally terminated. If we must empty the uterus it is wise, while this is being done, to begin our treatment for the elimination of the toxins. This may save valuable time. In most cases the patient will soon become conscious after the first convulsion, or so nearly conscious that she can swallow. She should immediately be given a dose of magnesium sulphate or calomel in order that the action of the bowels may be started as soon as possible. At the same time the remedy of choice should be given to control the convulsions. The best of these are chloroform, chloral, veratrum viride and morphin. I look upon chloroform as valuable only to hold the convulsions in check until the other remedies can take effect, but for this it must be used continuously and not intermittently as usually given. To give it only when the convulsion begins does not accomplish much, for the convulsion is over before the patient is under the effects of the chloroform. Chloral has unquestionably been most in favor for controlling the convulsions. It has not always accomplished what was expected of it, but this is often because it has not been used in large enough doses. The tendency to convulsions is hard to overcome, and when it exists the system will stand enormous doses. The small doses so frequently given are of no avail. I would give, as the smallest dose, twenty-five to thirty grains by the mouth, or fifty to sixty grains by the rectum, and repeat it in from one to four hours, if necessary.

Veratrum viride is a favorite drug with many. I have used it and felt that it was unquestionably effective. When the pulse is strong and of high tension the giving of frequently repeated doses of veratrum viride until the pulse is reduced to sixty or sixty-five beats per minute will often be effective. Morphin, which has always been condemned in cases of renal insufficiency, is being used by a few with surprising results. Stroganoff reports fifty-eight cases without a death. He gives one-fourth of a grain hypodermically immediately following the first convulsion, and repeats this once or twice at intervals of an hour according to the severity of the case. He then gives, in two hours, twenty to thirty grains of chloral, repeating the dose in from four to six hours as is needed to keep the patient drowsy for forty-eight hours. If the convulsions return he repeats the morphin. He looks upon eclampsia as a self-limited disease of forty-eight hours' duration, and considers that if the convulsions can be controlled and the heart sustained during this time the patient will recover.

In a recent copy of the *Glasgow Medical Journal* Weit is quoted as having used morphin in sixty cases with two deaths. The Rotunda Hospital, Dublin, is reported in the same *Journal* as having treated twenty-six cases with chloral and chloroform and lost eight cases. They also treated seventeen cases with morphin and lost three cases. These figures are most encouraging, and if further treatment by this method results as favorably much of the prejudice against morphin will be removed. As in all methods employed great care must be used and each case carefully studied. While using these remedies we must be just as energetic in our attempts to reestablish the functions of the various organs which can aid in eliminating the toxins present in the blood.

As has already been said one of the first remedies given should be a cathartic, my preference being a saturated solution of magnesium sulphate. An enema should be given as early as possible. It flushes the lower bowel, removing such toxins as may be there, and cleanses the mucous membrane so that it will the better absorb remedies if the patient cannot swallow them. Calomel is a good substitute if the salts cannot be obtained at once.

To promote the action of the skin the wet hot pack is frequently effective, but it may be slow to act or fail entirely. Pilocarpin is not generally advisable because of the danger of pulmonary edema. A valuable remedy to promote diaphoresis is the subcutaneous injection of salt solution, but its greatest value is that it produces diuresis. Ordinary diuretics are of no avail at such a time. Enough cannot be given to be effective, and they are more likely to prove an irritant to the kidneys if we give them. Water cannot be given in large enough quantities to be effective, but by using a saline solution subcutaneously we introduce into the system large quantities of fluid which will be rapidly absorbed, and cause, in most cases, profuse diaphoresis and diuresis. Flushing the bowels with such a solution and leaving a quantity in the colon to be absorbed is also beneficial.

Some advocate bleeding when the pulse is very full and strong. Others claim that just as good results can be obtained from veratrum viride. I have had no experience in blood-letting, though one case reported to-night had been bled before I saw her. After bleeding, the free use of salt solution used subcutaneously should be very effective. Intravenous injection of saline solution should be given cautiously, as there might be great danger of overdistention of the heart.

Among other remedies used are nitroglycerin and of late thyroid extract. These do not offer any special advantages not found in the remedies already mentioned, though the thyroid is especially recommended for its diuretic effect.

Within the past few months I have seen three cases of eclampsia which presented the most serious phases of this symptom complex.

A report of them may aid in emphasizing some of the points mentioned above. Two of the cases I did not see until after there had been convulsions. The third I had under my observation for three weeks previous to labor, but in spite of the greatest care she had convulsions, though not until after labor was terminated, which, considering the conditions found when the case was first seen, was more than I had dared to hope.

CASE 1.—Mrs. F. was seen in consultation with Drs. C. and W. She was twenty-seven years of age and had had one child. While carrying it she had edema of the legs and had noticed some puffiness of the face. She did not become pregnant again until four years later. The last menstruation was in September, 1901. The progress of pregnancy was apparently normal until April, 1902, when she began having severe headaches and also swelling of the ankles and face. The headaches became more severe until May 12, when she had a convulsion which was followed by three more in an hour. Under chloroform anesthesia the os was dilated manually by Dr. C., and a living child was delivered with forceps two hours after the first convulsion. After this the patient became conscious but in an hour had another convulsion, followed by nine more without the recovery of consciousness. The last one was twelve hours after delivery, and the patient did not again become conscious, dying eighteen hours later, or thirty-two hours after the first convulsion.

When I saw her, fourteen hours after the first convulsion, I found that she had been given two doses of a mixture containing chloral, bromide, hyoscyamus and cannabis indica per rectum. Chloroform was given at intervals. Hot packs failed to produce sweating and a hypodermic of polocarpin was given, also veratrum viride, after which there was profuse sweating. She had also been bled, but the amount withdrawn is not known. There had been no action of tne bowels or kidneys. No cathartic had been given. The patient was unconscious and had what proved to be her last convulsion while I was in the room. Because of the continual recurrence of the convulsions it was deemed best to give a hypodermic injection of morphin. Two lines of treatment were urged, one to obtain free catharsis and the other to use subcutaneous injections of salt solution. The cathartic was not given till the next day, or twenty-six hours after the first convulsion, and no result was obtained from it. Three pints of saline solution were given subcutaneously, but, with other treatment, failed to save the patient. There had been no examination of the urine until after the first convulsion when the patient was catheterized. The urine obtained was examined and showed a large quantity of albumin and casts.

CASE 2.—Mrs. K. was seen in consultation with Dr. S. who gave me the following facts: She was a multipara, having had one child and two miscarriages. The cause of these is not known. The child died when five days old. The previous pregnancies were apparently normal. In the present pregnancy the patient did not know just when she last menstruated, but thought she was about six and one half months pregnant. The pregnancy was apparently normal up to a short time previous to the onset of the convulsions, when she called her physician because of headaches. At this time he made his first examination of urine which showed the presence of albumin, and he immediately began treatment accordingly. Previously several attempts had been made to have urine sent for examination, but various combinations of circumstances prevented any being obtained.

About 9 o'clock on the morning of June 10 she had a convulsion and a second one at 11:30. I saw the case at 12:30. The patient was still unconscious from the last convulsion. Her pulse was 96 and was full and strong. She had been given one fourth of a grain of elaterium and one grain of calomel. Chloroform was administered in the attempt to prevent further convulsions. Immediate delivery of the child was advised and after careful preparation of the patient the os was dilated. At first it just barely admitted one finger. When dilated sufficiently to admit two fingers I found that the placenta was attached low down to the posterior wall of the uterus. The slightest efforts to dilate the os loosened the edge of the placenta, but the hemorrhage was controlled by pressing down the head from above the pubes so that it compressed the placenta. This made it more difficult to dilate the os but at the end of an hour I was able to apply forceps and deliver the child. The baby, about a seven months' child, was put into an incubator three hours after delivery but died four hours later.

The after-treatment of the case consisted of 20 grains of chloral every three hours and three doses of veratrum viride of 10 drops each at half-hour intervals, which reduced the frequency and volume of the pulse. The larger doses of chloral were not given because of the use of veratrum viride. One quart of saline solution was used subcutaneously. About six hours later a hypodermic injection of one fourth grain of morphin was given by her physician because of her restlessness. There were no drawbacks in the progress of the case and she made a good recovery.

CASE 3.—Mrs. A. was the wife of a physician. She had had one child, the pregnancy and labor being normal. I was asked to take charge of the case about three weeks before labor was expected. The pregnancy appeared normal up to three weeks before this, when swelling of the face and hands began. There had been swelling of the ankles before this. Examination of the urine showed albumin. When I first saw the patient her legs were swollen to nearly double their normal size. The face and hands were swollen and there was some edema of the labia. There was also headache and some difficulty in seeing. The quantity of urine, for twenty-four hours, was a pint and a half. Examination of it showed one half volume of albumin, hyalin and granular casts, leukocytes and some red blood-cells. That the history may not be too long the report of the many examinations will be omitted. The patient's diet was restricted to milk with only slight variations; she was given large quantities of lithia water to drink, the bowels were kept free, and a warm bath was given each day, with the result that there was almost complete relief from headache and the swelling of the face and hands and legs diminished markedly. The urine was increased to three and sometimes four pints a day, casts and red blood-cells almost entirely disappeared and albumin decreased to about one third volume. At the beginning of the treatment improvement came so slowly that the advisability of producing labor was considered. This, however, was not done, and labor began about three weeks after treatment was begun. It lasted seventeen hours and was normal. Chloroform was used, but not extensively, during the last three hours. There was no headache or other bad symptoms during labor, though immediately following there was some dizziness. About four hours later headache began. Fearing that this might be the forerunner of convulsions the patient was given ten grains of chloral and this was repeated in a couple of hours. Seven hours after labor her temperature was normal, and on catheterizing about one pint of urine was obtained. Examination of this specimen showed an increase of albumin to about one half volume. In response to a telephone message, that the headache persisted, a small dose of phenacetin was suggested, which almost instantly stopped the headache. For an hour the patient felt very well, when without warning she had a convulsion. Chloroform was given by the doctor, and when I saw her soon after she was given twenty-five grains of chloral by the mouth. Though the bowels had moved freely before labor half an ounce of magnesium sulphate was given by the mouth. Chloroform was given till the chloral had had time to act. She slept two hours and on rousing was given ten grains of chloral by the stomach and went to sleep again but wakened in an hour and had another convulsion. Fifty grains of chloral were given per rectum. In spite of this she was very restless and one eighth grain of morphin was given hypodermically and repeated in three hours, keeping the patient quiet for several hours. Immediately following the first convulsion a saline solution was given subcutaneously. It was given at intervals till she had had four quarts. A wet hot pack was used but accomplished very little. After absorption of about three quarts of saline solution she began to perspire very freely. Twelve hours after she was first catheterized another pint of urine was obtained. She was allowed to go so long because she was quiet and it did not seem wise to disturb her. An enema had been given earlier in the night and the bowels began moving freely. From this time on the patient's condition improved and she made a good recovery.

I have given the details of this case more fully than the others because I had greater opportunity of watching it both before and after the convulsions.

In comparing the three cases it will be seen that each presented early symptoms of danger. In the first case no examination of the urine had been made previous to the first convulsion but there were other symptoms which were very suggestive. In Cases 2 and 3 the first examination of the urine was not over three weeks before the occurrence of the convulsions. These cases illustrate the need of an early examination of the urine in every instance. Whether Case 1 would have been saved if this had been done we cannot say. Her chances would have been improved. It is certain too that the chances of Case 3 would have been very much decreased if she had not had vigorous treatment for the three weeks preceding labor.

The treatment of Cases 2 and 3 was the same as Case 2 after delivery. In each very free action of the bowels was obtained as soon as possible after the first convulsion, while in Case 1 nothing was given for twenty-four hours and no action was obtained. In Cases 2 and 3 sufficient chloral and morphin were given early to keep the patients under control and salt solution was given so that the kidneys and skin acted freely. It does not follow that if these same means had been used early in Case 1 there would have been fewer convulsions or that the patient would not have died, for cases may die in spite of the most vigorous treatment.

I have compared these cases, not to criticize or commend the treatment used in either, but to emphasize if possible the need of thorough treatment at the very beginning of convulsions. My experience not only in these cases, but in others which I cannot take time to report, is that there is too great fear of overdoing the treatment of eclampsia with the result that far too little is done. It is only large doses that will control convulsions, and they must be given promptly and fearlessly. This is one of the points I wish to urge as strongly as possible and which has led me to present this paper.

There is another point that is even more important but only because, by enforcing it before the convulsions occur, the first one mentioned will seldom be needed, that is prophylaxis. In every paper I have written on any obstetric subject I have urged that greater attention be given to obstetric cases in the preparation for confinement. The patients themselves do not appreciate the value of it and they never will if they are not instructed by their physicians, for so many cases terminate normally that a false feeling of security is the result. In no single case can we predict that we shall escape without a convulsion, therefore in no one case are we excusable if we leave undone those things which will in all probability warn us of impending danger. I realize that it is hard to get specimens of urine to examine and to always determine positively from them that danger exists. I realize that, in spite of watchfulness, eclampsia sometimes occurs without any apparent premonitory symptoms. I have had such experiences. These experiences, however, should lead us to be even more thorough in our cases or we had better not undertake them. A mere examination of the urine for albumin is not sufficient, for convulsions may occur when it has been absent, but a careful study of each case will seldom fail to give us some warning, even though slight, in time to adopt effective prophylactic measure.

The following case seen with Dr. G., since reading this paper further illustrates the treatment recommended. The patient had been under the doctor's observation for some time because of albuminuria. About the end of the seventh month she had a convulsion, followed by another in half an hour. A hypodermic injection of veratrum viride reduced the pulse from 130 to 80. When I saw her an hour and a half later a hypodermic injection of morphine was given. Urine obtained by catheterization became solid on boiling. Under chloroform the os was manually dilated and the child, which was dead, was delivered. Two quarts of saline solution were given subcutaneously and medical treatment as above outlined was carried out. There were no more convulsions. Forty-eight hours later there was a serious failure of the heart's action, which was overcome after a few hours, but with this exception the patient made an uninterrupted recovery.

REFERENCES.

Hare's Progressive Medicine.
Gould's Year-Book of Medicine and Surgery,
Jewett's Text-Book of Obstetrics,
American Text-Book of Obstetrics,
Practical Obstetrics by Grandin and Jarman,
Text-book of Obstetrics by Hirst,
Practical Obstetrics by Reynolds and Newell.

—*Cleveland Medical Journal.*

### BUFFALO LITHIA WATER AS A SOLVENT.

BY J. SIMONIDES GRANT, M.D., NEW YORK.

Only such natural waters as possess therapeutic properties far above the ordinary break the bound of local environment and find their way into the outer world. And the extent to which such spread of popularity may go is a fair standard by which to gauge the therapeutic merits of a natural mineral water.

Measured by this standard Buffalo Lithia Water is far ahead of all other medicinal waters in the estimation of the medical profession; not only is it known and prescribed by many physicians throughout all the Americas, but it is also used in many foreign countries.

Buffalo Lithia Water was first brought to my notice by Dr. J. S. Todd, Professor of Materia Medica and Therapeutics in the Atlanta Medical College, in consultation in a severe case of pneumonia. Prof. Todd suggested milk and Buffalo Lithia Water equal parts alternated with equal parts of whiskey and Buffalo Lithia Water every hour or two pro re nata. I adopted his treatment with the most gratifying results; and it has been to a great extent a regular routine with me ever since. Under this treatment I always find a decided lessening of the febrile movement which I attribute to the pronounced solvent influence of the Buffalo Lithia Water on the kidneys. It undoubtedly stimulates those organs and increases their power of eliminating toxic elements from the blood.

In acute albuminuria of pregnancy experience has taught me to regard the solvent properties of Buffalo Lithia Water as a specific. It is my practice to guard my patients against this too frequent complication in pregnancy by a timely use of this water.

I have them drink it freely from the beginning of the sixth or seventh month to its conclusion. This treatment, with proper regulation of diet and proper hygiene, insures a safe and easy delivery and leaves the patient in proper condition to meet all the requirements of motherhood.

If, however, the albuminuria be pronounced and persistent, as is frequently the case when I am called in the last few weeks of pregnancy, I push the Buffalo Lithia Water to the limit, allowing my patient as much of it as she can take without positive discomfort. Its solvent properties rapidly eliminate the urea and other morbid elements from the blood, and relieve vomiting and other symptoms of gastro-intestinal disturbances. There is also a rapid diminution of the intensity of renal inflammation, a promotion of resolution and restoration of the secretory functions.

In severe cases, where there are pronounced symptoms of uremic poisoning, coma or eclampsia, the intravenous administration of Buffalo Lithia Water, with sodium chloride added, will frequently save the life of the patient, even when all other remedies and measures have failed.

And this reminds us that Buffalo Lithia Water, on account of its absolute purity and smoothness, is a most desirable menstruum for the intravenous administration of saline or normal salt solutions wherever and whenever indicated or necessary.

The therapeutic value of Buffalo Lithia Water in so-called skin diseases cannot be overestimated. I say "so-called" because I regard this class of ailments as symptoms rather than diseases. They are usually indications of defective metabolism, or functional derangements of certain vital organs. This statement is supported by no less an authority than one of our most famous Dermatologists, Prof. Geo. Henry Fox, who says that this class of ailments belongs to the domain of the general practitioner.

But it is in that class of skin lesions, such as the eczemas, acnes, erythemas, etc., due to an uric acid diathesis that I have found the solvent properties of Buffalo Lithia Water of the greatest value. That it neutralizes and eliminates this acid all physicians who have used it and carefully noted its results (by urinary tests, etc.) are agreed. And it is logical to suppose that this result is largely due to the fact that by its solvent power this water materially increases the metabolic forces and prevents the contributory or prime cause in skin lesions; in other words Buffalo Lithia Water not only removes the cause in this class of ailments but it also eliminates (through the kidneys and other excretory organs) the débris or toxins which are invariably present.

The same theory applies to the therapeutic solvent effects of Buffalo Lithia Water in rheumatism and gout; the former supposed to be due to an excess of uric acid, the latter to too much lactic acid. The extraordinary value of this water in both these affections is attested by a large majority of cures, after other methods had failed. Buffalo Lithia Water also gives most excellent results in neurasthenia or nervous prostration; more especially when that condition is due to mental strain or overwork.

In diseases of the alimentary tract, such as gastritis, acute and chronic, intestinal indigestion, colitis, etc., Buffalo Lithia Water gives much satisfaction. It prevents the formation of noxious gases and inhibits the development of toxic organism.

I have also found it equally effective in diseases of the urinary bladder and its mucous lined connections. The great solvent properties of Buffalo Lithia Water prevent the formation of renal or urinary calculi, and also greatly facilitate their disintegration and expulsion if already formed.

Its solvent and eliminating properties seem to be due to the peculiar combination of the lithium and other alkaline carbonates which it contains, making Buffalo Lithia Water the most powerful solvent of uric acid of which we have any knowledge.

Not only is uric acid the nucleus of such formations, but it also enters largely into the various layers of their superstructure; a fact which accounts for the rapid solution, breaking up, disintegration and elimination of such formations under the administration of Buffalo Lithia Water. In like manner this water seems to dissolve and eliminate the lacto-phosphatic deposits peculiar to gout.

The tonic and solvent effects of Buffalo Lithia Water seem to be due to the fact that its composition is approximately that of the serum of the blood (Shoemaker); therefore, it becomes at once identical with the blood, and is more easily assimilated.

In conclusion I desire to impress upon those who have failed to obtain satisfactory results from the employment of Buffalo Lithia Water, and those who have not given it a trial in practice, one important fact, and that is, that the water must be taken regularly and systematically and for a reasonable length of time to insure its full effect and obtain the best results.

In chronic conditions of long standing the metamorphosis has, as a rule, been slow and extended over a considerable period; therefore, it stands to reason that Buffalo Lithia Water must be taken for a corresponding period to secure its full therapeutic effects.

---

### DIET IN SUMMER COMPLAINT.

BY WILLIAM F. WAUGH, M.D., CHICAGO, ILL.

One of the most difficult of tasks is to persuade the panic-stricken young mother that her sick child does not require constant feeding. It seems so reasonable that the more the baby is weakened by his illness, the more he needs his vitality to be restored by food. But putting food into the stomach is not feeding the child, even if the food is not vomited or purged away. In fact it may be poisoning the infant in place of nourishing it.

The child with cholera infantum comes to us with its stomach and bowels containing more or less poisonous material. Absorbed into its blood its effects are manifested in fever, restlessness, irritability of the gastro-intestinal branches of the pneumogastric nerve, insomnia, headache, perhaps convulsions. Obviously, the first indication is to sweep out of the alimentary canal this toxic matter and render this tube as nearly surgically clean as possible. Mercury with chalk $^1/_{12}$ grain every quarter-hour for five doses, followed by alkaline syrup of rhubarb, a teaspoonful every two hours, fulfils the first indication.

Wash out the stomach and colon with warm water, containing $\frac{1}{4}$ to 1 grain of zinc sulphocarbolate in each ounce, and give $\frac{1}{4}$ to $\frac{1}{2}$ grain of this salt every half-hour until the stools are free from odor; and the second indication is met. If the stomach is irritable add to each dose 1-grain each of bismuth subnitrate and saccharated pepsin, to soothe the organ.

The third indication is to restore the secretions, normal in quality, of the stomach and bowels. For this we give emetine $\frac{1}{250}$ grain, juglandin $\frac{1}{67}$ grain, or rhein $\frac{1}{34}$ grain every hour, with excellent effect.

Then, and not till then, are we ready to give the child food. At first a few ounces of normal salt solution, quite warm, should be gently drained into the colon. Let the stomach rest. If drink is craved give a teaspoonful of water as hot as can be sipped. Do not repeat within half an hour—constant drinking excites unconquerable thirst. Hot baths allow enough absorption through the skin to notably increase the weight of the body. After twelve hours' abstinence, let the child have teaspoonful doses of coffee—pure, with cream, sugar, or both, as best agrees. Of course sugar is bad, but I have known a child retain sweetened coffee when the sugarless was vomited. The raw white of an egg in cold water comes next, then rice, barley or toast water; the plain, strained soups, cold consommé, then raw beef or oysters, grated, and gradually other foods. In all cases teaspoonful doses of food, not less than two hours apart, until convalescence, appetite and digestive capacity have returned. When fermentation is no longer a factor, begin to give freshly pressed fruit juices, at first similarly small doses.

No milk!

Pepsin is worth more as a sedative of the irritated stomach than as a digestant.

Total abstinence from all food and drink for twenty-four hours has stopped a vomiting that resisted every other remedy.

It is not enough to give directions to the family. No consideration will prevent relatives poking food down the throat of a sick child the moment the doctor's back is turned. If there is no determined old aunt who can be relied upon, get a trusty and arbitrary trained nurse, or stay yourself, if the case is a bad one. Otherwise the child will die.

We have not yet developed civilization to the point at which the vendors of impure milk are lynched, and until then it is best to quit using it for infants in hot weather. There are excellent brands of condensed milk, and better still, of cream, to be had anywhere, and if milk be considered absolutely essential, these should be employed.

But is milk necessary ?—*Courier of Medicine.*

---

### PUERPERAL SEPSIS.

Edward P. Davis states in the *Philadelphia Medical Journal* for May 23, that the method of treatment of proven value in this condition is, first, to thoroughly cleanse the uterus and vagina, using every precaution to avoid opening channels for fresh absorption of septic material and removing gently and thoroughly possible infective material within the uterus. Cold to the abdomen over the uterus, combined with counterirritation by a turpentine stupe large enough to extend from the pubes to the umbilicus, over which an icebag is placed, is, in his experience, the best method for combating pain in the abdomen in these cases. For the prompt and thorough emptying of the intestines salts are of value, but he prefers the compound cathartic pill. Medication should be limited to those drugs which act as tonics and stimulants to the nervous system and produce contraction of the uterus. Strychnin and ergot are most valuable and should be given together every four to six hours until distinct improvement has occurred. Alcohol is the best internal antiseptic, and the only limit to its administration here is the patient's capacity to absorb it without intoxication. All septic patients must be liberally fed, and the use of normal salt solution by bowel or hypodermoclysis should be followed in all serious cases. Methods of treatment which must still be considered as experimental are the use of antistreptococcic serum, the use of nuclein to produce increased leukocytosis, the use Crede's silver ointment, and the intravenous injection of formalin. Methods of treatment which he considered injurious are, first, to give a septic patient drugs to reduce the temperature, as no drug controls the temperature without depressing the patient. He prefers the use of cold and the administration of alcohol or hot sponging followed by the use of alcohol. Another source of useless and injurious drugging arises from the anxiety of the physician to stimulate the heart. There are times in septic cases in which the value of digitalis becomes evident, but its use is seldom necessary if the patient's nutrition is properly secured. Drugs are also used injuriously in these cases when an effort is made to check the purgation which nature often sets up, and it is also seldom necessary to give drugs to procure sleep in these cases. Dr. Davis strongly emphasizes the mistake of repeated intrauterine manipulation, as well as the dangerous results following the use of bichlorid of mercury within the womb. After the uterus is emptied lysol 1 per cent., creolin 1 per cent., or normal salt solution, should be chosen. He has never seen nor known of a case of iodoform poisoning from the use of iodoform gauze within the puerperal uterus.—*Cleveland Medical Journal.*

---

### DEATH OF DR. I. N. LOVE.

We were shocked to learn of the sudden death, on June 18th, of Dr. Isaac Newton Love, of New York City, formerly of St. Louis. Dr. Love had made a short visit to Europe. The steamer on which he returned had reached the pier, and Dr. Love was in the midst of an address to his fellow-passengers, expressing his joy at the safe arrival of all at home, when he was smitten with apoplexy and instantly expired.

Dr. Love was in the prime of life, vigorous of body and alert of mind. He was one of the best-known men in the profession of this country. A nephew of the celebrated St. Louis surgeon, Dr. Hodgen, under whom he studied, Dr. Love entered the profession under the best auspices. He achieved marked success as a practitioner in St. Louis, and gradually was led to make a special study and practice of pediatrics. In this branch he was eminently successful. The deceased was a notably cordial, genial man, and his kind manner peculiarly fitted him to deal with children. He was prominent in the public and organized life of the profession. In the medical societies of St. Louis, of the State of Missouri, of the Mississippi Valley, and in the American Medical Association he was a constant contributor of suggestive, practical papers, taking his part also in the discussions. He had served in the board of trustees of the American Medical Association.

Dr. Love was widely known as a medical journalist. He was the founder and editor of the *Medical Mirror*, a periodical imbued with his personality. To the columns of this journal he had contributed a large number of editorial articles, interesting from the literary and personal as well as medical point of view.—*Medical Bulletin.*

